# Understanding the Red Shift in the Absorption Spectrum of the FAD Cofactor in ClCry4 Protein

**DOI:** 10.1021/acs.jpcb.4c00710

**Published:** 2024-05-28

**Authors:** Katarina Kretschmer, Anders Frederiksen, Peter Reinholdt, Jacob Kongsted, Ilia A. Solov’yov

**Affiliations:** † Institute of Physics, Carl von Ossietzky Universität Oldenburg, Carl-von-Ossietzky Str. 9-11, 26129 Oldenburg, Germany; ‡ Department of Physics, Chemistry and Pharmacy, University of Southern Denmark, DK 5230 Odense, Denmark; § Research Centre for Neurosensory Science, Carl von Ossietzky Universität Oldenburg, Carl-von-Ossietzky Str. 9-11, 26129 Oldenburg, Germany; ∥ Center for Nanoscale Dynamics (CENAD), Carl von Ossietzky Universität Oldenburg, Ammerländer Heerstr. 114-118, 26129 Oldenburg, Germany

## Abstract

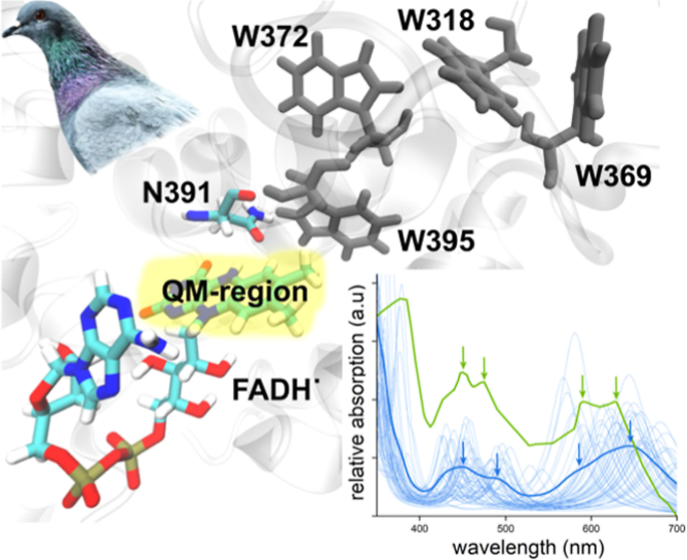

It is still a puzzle that has not been entirely solved how migratory birds utilize the Earth’s magnetic field for biannual migration. The most consistent explanation thus far is rooted in the modulation of the biological function of the cryptochrome 4 (Cry4) protein by an external magnetic field. This phenomenon is closely linked with the flavin adenine dinucleotide (FAD) cofactor that is noncovalently bound in the protein. Cry4 is activated by blue light, which is absorbed by the FAD cofactor. Subsequent electron and proton transfers trigger radical pair formation in the protein, which is sensitive to the external magnetic field. An important long-lasting redox state of the FAD cofactor is the signaling (FADH^•^) state, which is present after the transient electron transfer steps have been completed. Recent experimental efforts succeeded in crystallizing the Cry4 protein from *Columbia livia* (ClCry4) with all of the important residues needed for protein photoreduction. This specific crystallization of Cry4 protein so far is the only avian cryptochrome crystal structure available, which, however, has great similarity to the Cry4 proteins of night migratory birds. The previous experimental studies of the ClCry4 protein included the absorption properties of the protein in its different redox states. The absorption spectrum of the FADH^•^ state demonstrated a peculiar red shift compared to the photoabsorption properties of the FAD cofactor in its FADH^•^ state in other Cry proteins from other species. The aim of this study is to understand this red shift by employing the tools of computational microscopy and, in particular, a QM/MM approach that relies on the polarizable embedding approximation.

## Introduction

It has long been known that migrating birds have an internal magnetic compass that helps them navigate during the biannual migration periods. Particularly, they utilize the geomagnetic field for this purpose.^1−3^ The cryptochrome 4 (Cry4) protein has been proposed as the specific receptor to endow migratory birds with the enigmatic sense.^4,5^ Later, it was shown that activation of the protein could be influenced by the weak magnetic fields, comparable with the geomagnetic field.^6^

The only logical explanation so far for how the geomagnetic field can influence the chemical processes within the Cry4 protein is through the radical pair (RP) mechanism, which occurs in the protein following a blue-light-induced electron transfer (ET).^7−11^ An RP is a short-lived reaction intermediate that contains two radicals. Their unpaired electron spins can be either antiparallel ↑↓ (singlet state) or parallel ↑↑ (triplet state).^6,12,13^ In Cry4, one radical is formed from the flavin adenine dinucleotide (FAD) cofactor, which is a cofactor that can bind noncovalently inside the protein. The other radical is formed from the tryptophan (W) amino acid residue in Cry4, where one of the four tryptophans of the well-conserved Trp-tetrad is involved.^8−10,14−16^

Previous studies indicated that the FAD cofactor inside the Cry protein needs to be activated through light. Light absorption by the FAD cofactor initiates ET inside the Cry4 protein. Starting with a nearby tryptophan residue (W395), an electron is transferred through a Trp-tetrad in cryptochrome to form the radical pair [FAD^•–^ Trp395H^•+^] = [FAD^•–^ TrpH^•+^]. The singlet and triplet states of this RP can be converted into the FADH^•^ state through additional FAD^•+^ protonation, leading to Cry4 entering the signaling state. This FADH^•^ state is either further reduced or results in another radical pair [FADH^•^ O_2_^•–^] that was speculated to be magnetic field sensitive.^17−21^ In this study, the FAD cofactor is considered to be in the FADH^•^ state.

In a previous study, the Cry4 protein from pigeon (*Columbia livia*) (ClCry4) was investigated experimentally.^22^ The study delivered the crystal structure of the protein with the internally bound FAD. For the crystal structure of the ClCry4 protein, different transient absorption spectra of the protein were analyzed, and certain peculiarities were recorded.^22^ The absorption spectrum of the FAD cofactor in the FADH^•^ state showed an unusual shift at a wavelength of ∼600 nm compared to other cryptochromes, e.g., cryptochrome 1 from *Arabidopsis thaliana* (AtCry1). In the case of the ClCry4 protein, the FADH^•^ state might be reached through protonation from the surrounding solvent. Past experimental investigations have confirmed the presence of the FADH^•^ cofactor in ClCry4 and suggested that this state is representative of the signaling state of the protein.^10,22−24^ Computational studies^23,25^ have furthermore indicated that upon activation, ClCry4 opens a molecular pocket, which allows for more water molecules to get closer to the FAD cofactor, possibly paving the way for its protonation. In AtCry1, a different mechanism is at play. Here, the D396 residue, strategically placed near the FAD^•–^ cofactor, is expected to act as the H^+^ source. Earlier studies corroborated this finding.^7,26,27^ In the present investigation, we aim to understand the differences in the absorption spectral shift of ClCry4 protein and rationalize it through comparison with other species.

We explain the observed red shift through calculations of the absorption spectrum of the FADH^•^ cofactor in the ClCry4 protein using the polarizable embedding (PE) method.^28^ In particular, we confirm that the asparagine (N391) residue plays a key role in the red shift, as has been speculated in a previous study.^22^

## Methods

The methods used for the present study were largely similar to those employed previously for studying photoabsorption of Cry proteins from different species.^29,30^

**Figure 1:**
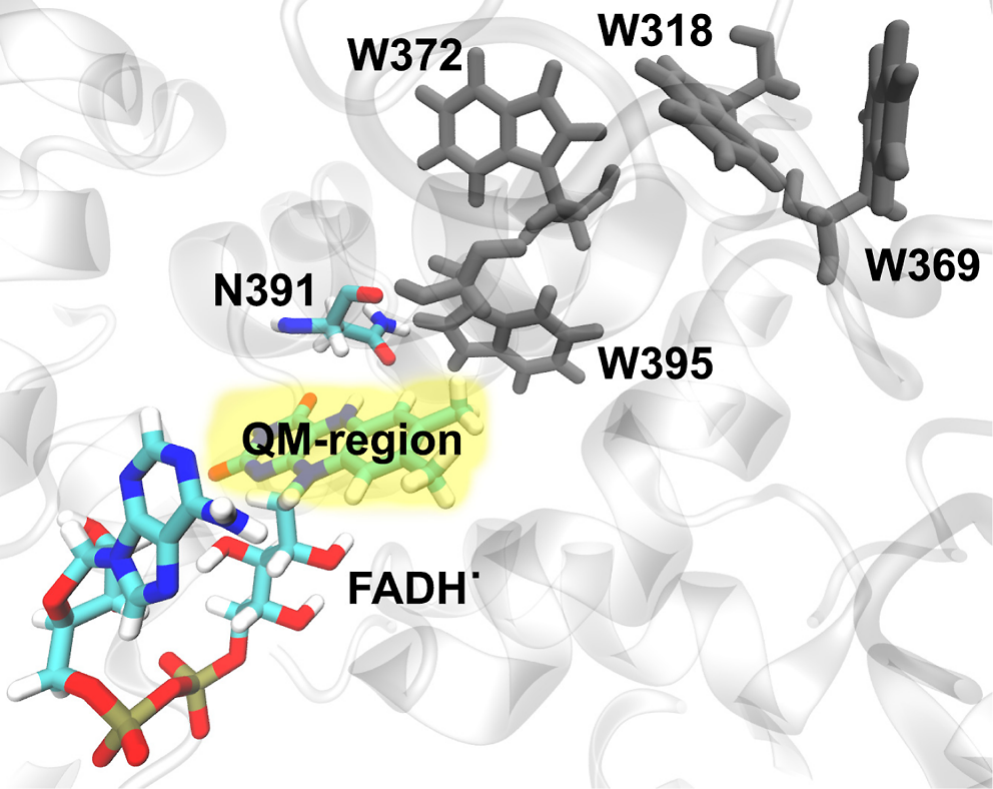
FADH^•^ cofactor and the residues W395, W372, W318, and W369 involved in the ET process inside of the ClCry4 protein. The residue N391, specific for the ClCry4 protein, is indicated. The atoms inside the marked QM-region were treated quantum mechanically.

The crystal structure of the protein was taken from the protein data bank (6PU0).^22^ Earlier studies^23^ suggested specific protonation states of the protein that were adopted here. Interatomic interactions were modeled with the CHARMM36 force field with CMAP corrections.^31−37^ The parameters for the FADH^•^ cofactor were also determined earlier.^8,38,39^ The atomistic model of the ClCry4 protein was simulated in a water box of dimensions ∼96.73 Å × 96.73 Å × 101.56 Å, assuming periodic boundary conditions with sodium and chlorine ions added to neutralize the system and a physiological NaCl concentration of 50 mM. The temperature of the system was set to 310 K. Figure 1 shows the solvated ClCry4 protein with a FADH^•^ cofactor. Additionally, residue N391, which appears close to the FADH^•^ cofactor, is indicated. The residues W395, W372, W318, and W369, involved in the ET transfer in the ClCry4 protein, are also shown. The system was simulated first in the *NPT* ensemble for equilibration purposes (duration 350 ns), and afterward, production simulations of 500 ns duration in *NVT* were carried out. Simulations were carried out using the NAMD software.^40,41^ Production simulation used for analysis was used to generate snapshots of the system at the time instances separated by 10 ns (50 snapshots in total) for the quantum calculations. To simulate the N391D mutation inside the ClCry4 protein, another production simulation was set up (duration 150 ns, 15 snapshots in total). The starting configuration for the MD simulation of the N391D mutation was taken after 500 ns long production simulation of the wild-type ClCry4 protein.

The photoabsorption spectra of the FADH^•^ cofactor were computed using the PE method,^28,42−44^ which permits calculating the absorption spectra with account of the ClCry4 protein scaffold. The method combines quantum mechanics (QM) with molecular mechanics (QM/MM).^42^ The flavin part of the FAD cofactor in its FADH^•^ state was treated quantum mechanically and was denoted as the quantum region (QM-region). Only the flavin moiety of the FADH^•^ cofactor was included in the QM region. The tryptophan close to the FAD cofactor of cryptochrome (W400 in AtCry1 and W395 in ClCry4) rapidly becomes cationic upon FAD photoexcitation, while the FAD cofactor acquires its FAD^•–^ anionic state. The charge on the tryptophan migrates through the chain of conserved tryptophans (triad in AtCry1 and tetrad in ClCry4) until the terminal tryptophan (W324 in AtCry1 and W369 in ClCry4) becomes positively charged. After some time, the FAD^•–^ cofactor accepts an additional proton, yielding the FADH^•^ state.^22^ The terminal positively charged tryptophan on the protein periphery is also expected to deprotonate,^9^ leaving the protein in the long-lived signaling state. The rest of the protein, water, and ions were treated as polarizable potentials, which interact with the QM region (environment region). The components of the environment were modeled through polarizabilities, point charges, and dipole and quadrupole moments surrounding the QM region. To further account for the influence of the environment region on the QM region, the polarizability of all atoms inside the environment region was calculated. For calculation of the multipoles and the polarizability, the LoProp approach was utilized.^45^ Point charges and dipole and quadrupole moments of the environment region were estimated through density functional theory (DFT) calculations using the 6-31+G* basis set. The hybrid functional PBE0 was used for the calculations. All amino acids in the protein were treated as closed shell, while the flavin part of the FADH^•^ cofactor was treated as an open shell fragment. The absorption spectra of the FADH^•^ cofactor inside the ClCry4 protein were computed using the CAM-B3LYP method, where PE was used to estimate the influence of the environment on the QM region.^42^ Linear response theory was employed to compute the photoabsorption spectra. The computed transitions were assigned peaks that were broadened with a Lorentzian function, assuming a full width at half-maximum of 0.0037 hartree. The calculations of the absorption spectra were carried out using the Dalton software.^46^

## Results and Discussion

**Figure 2:**
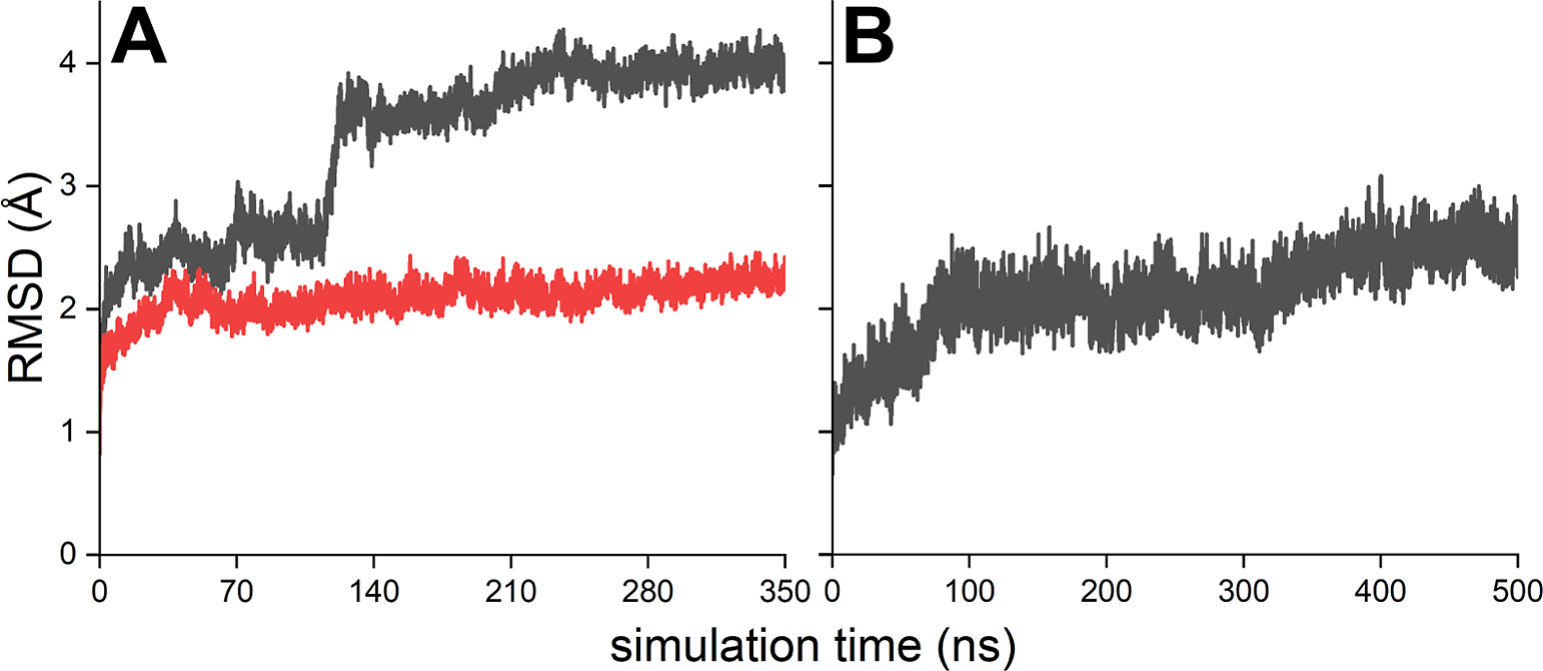
Time evolution of the rmsd of the ClCry4 protein backbone during the equilibration (*NPT*) (A) and the production (*NVT*) (B) simulations computed relative to the starting protein configuration in the respective simulations. For the *NPT* simulation, the rmsd of the protein and the FADH^•^ cofactor was plotted with (black line) and without (red line) the phosphate binding loop residues 220–245. The rmsd time evolution for the *NVT* simulation is shown for the complete protein backbone.

The time evolution of the root mean squared displacement (rmsd) of the protein was used to justify whether the simulated ClCry4 structure reached equilibrium, and this is shown in Figure 2. Here, it can be seen that the dominantly flexible region of ClCry4 is the phosphate binding loop composed of residues 220–245; flexibility is typical for the phosphate binding loop when the FAD cofactor is in the signaling FADH^•^ confirmation.^23^ The rmsd for the production simulation was also calculated to ensure that no notable changes in the proton structure remain (Figure 2B).

**Figure 3:**
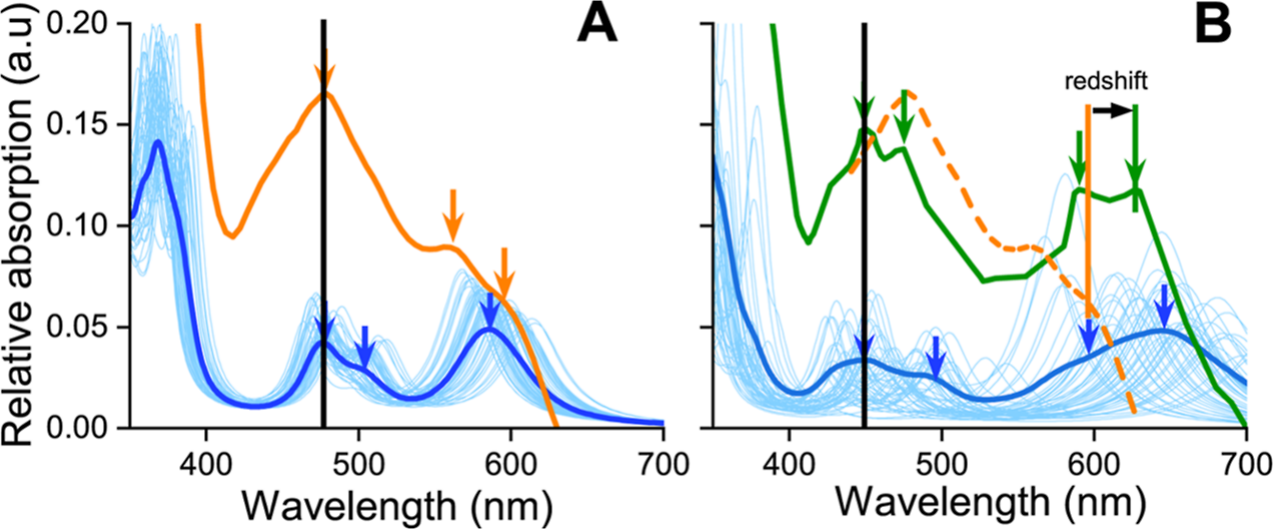
Photoabsorption spectra computed for the FADH^•^ cofactor inside AtCry1 (A) and ClCry4 (B). The experimental absorption spectrum for the FADH^•^ cofactor inside the AtCry1 (orange) and ClCry4 protein (green) was measured in previous studies.^22,26^ The computational photoabsorption spectra for AtCry1 (blue) were reused from a previous study.^30^ The thin lines represent the computed absorption spectra obtained for the different snapshots, while the solid lines represent the averaged absorption spectra over all snapshots for the AtCry1 and ClCry4 proteins, respectively. The arrows represent the pronounced peaks in the calculated and experimental absorption spectra of both proteins. The black vertical lines mark the peaks in both experimentally obtained absorption spectra, to which the computationally obtained absorption spectra for AtCry1 and ClCry4 were red-shifted. The green and orange vertical lines in (B) emphasize the experimentally observed red shift inside the ClCry4 protein compared to the absorption spectrum of AtCry1. The orange dashed line features a fragment of the AtCry1 absorption spectrum from (A).^22^

Photoabsorption spectra were calculated for the FADH^•^ cofactor in the ClCry4 protein for a number of snapshots. The corresponding transition dipole moments and the transition wavelengths of the QM-region were calculated. The computed absorption spectra from the TD-DFT calculations were compared with the absorption spectra of the AtCry1 protein from a previous study,^26^ as shown in Figure 3A. The results of the calculations obtained for the ClCry4 protein were also compared with the experimental results for the ClCry4 protein^22^ (see Figure 3B). The results of the calculations for the AtCry1 protein (see Figure 3A) were rescaled such that the amplitudes of the spectra became comparable with the experimental data. Additionally, the calculations were red-shifted 96 nm to match the position of the peak at 480 nm. In the case of the ClCry4 protein calculations (see Figure 3B), a red shift of 60 nm was applied to match the position of the peak at 450 nm. Figure S1 in the Supporting Information shows the computed photoabsorption spectra for the ClCry4 and AtCry1 proteins employing the TD-DFT approach. Here, the spectra were not assigned any additional shift to compare the experimental spectra with the computed ones.^22,26^ The figure shows a red shift of the first absorption peak of about 0.4 eV for FADH^•^ when embedded in ClCry4 compared to the first peak when it is embedded in AtCry1, which is similar to the expected red shift from experiments depicted in Figure 3.

Comparison of the photoabsorption spectrum for the ClCry4 protein (see Figure 3B) with the one for AtCry1 protein (see Figure 3A) reveals an experimental red shift of ∼20 nm in the region of ∼570 nm. Remarkably, this red shift is present in the experimental and the calculated absorption spectra for both proteins.

**Figure 4:**
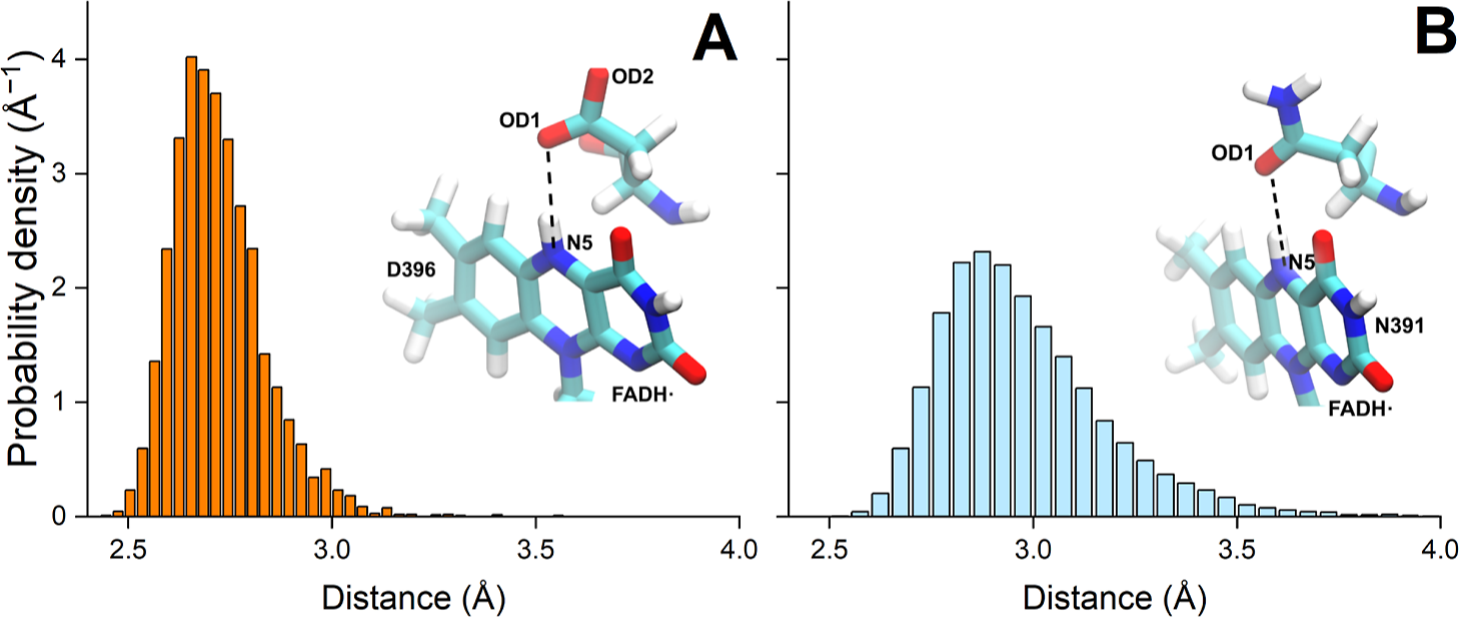
Normalized probability density distribution ρ(*r*) of the sampled distances between the flavin part of the FADH^•^ cofactor (N5 atom) and the oxygen atoms OD1 and OD2 of the D396 residue in the AtCry1 protein (A) and the flavin part of the FADH^•^ cofactor (N5 atom) and the OD1 atom of the N391 residue in the ClCry4 protein (B).

The molecular environment surrounding the FAD-cofactor in the ClCry4 protein includes the N391 residue, which was also suggested previously to influence the activation of the ClCry4 protein.^22^ The hydrogen bond length between the N391 residue and the FADH^•^ cofactor in the ClCry4 protein was calculated (see Figure 4B). Similar analysis was carried out for the H-bond between the D396 residue and the FADH^•^ cofactor in the AtCry1 protein in order to spot a difference between these two bonds (see Figure 4A); here, it is worth mentioning that the D396 residue in AtCry1 and the N391 residue in ClCry4 are spatially close to the corresponding FADH^•^ cofactor. The distances between the nitrogen (N5) atom of the FADH^•^ cofactor and both oxygen (OD1 and OD2) atoms of the D396 residue for the AtCry1 protein and the distance between the N5 atom of the FADH^•^ cofactor and the OD1 atom of the N391 residue for the ClCry4 protein were determined (see Figure 4). Normalized probability density distributions for the computed distances show that for both proteins, the H-bond between the FADH^•^ cofactor and the respective residue appears in both the AtCry1 protein and the ClCry4 protein might be reasonable since a distance from 2.7 and 3.3 Å is expected for protein hydrogen bonds.^47^ In the ClCry4 protein, the H-bond length is distributed at around 2.9 Å, while in AtCry1, it is around 2.7 Å. In the case of the AtCry1 protein, the distances between the N5 atom and both oxygen atoms were determined independently and compared with each other. The result in Figure 4A shows the distance between the N5 atom of the FADH^•^ cofactor and the closest oxygen atom from the D396 residue. The figure shows that the distance hardly exceeds 3.0 Å, while the probability distribution for the H-bond length case of ClCry4 has a longer tail, suggesting a less stable H-bond in the case of the ClCry4 protein.

**Figure 5:**
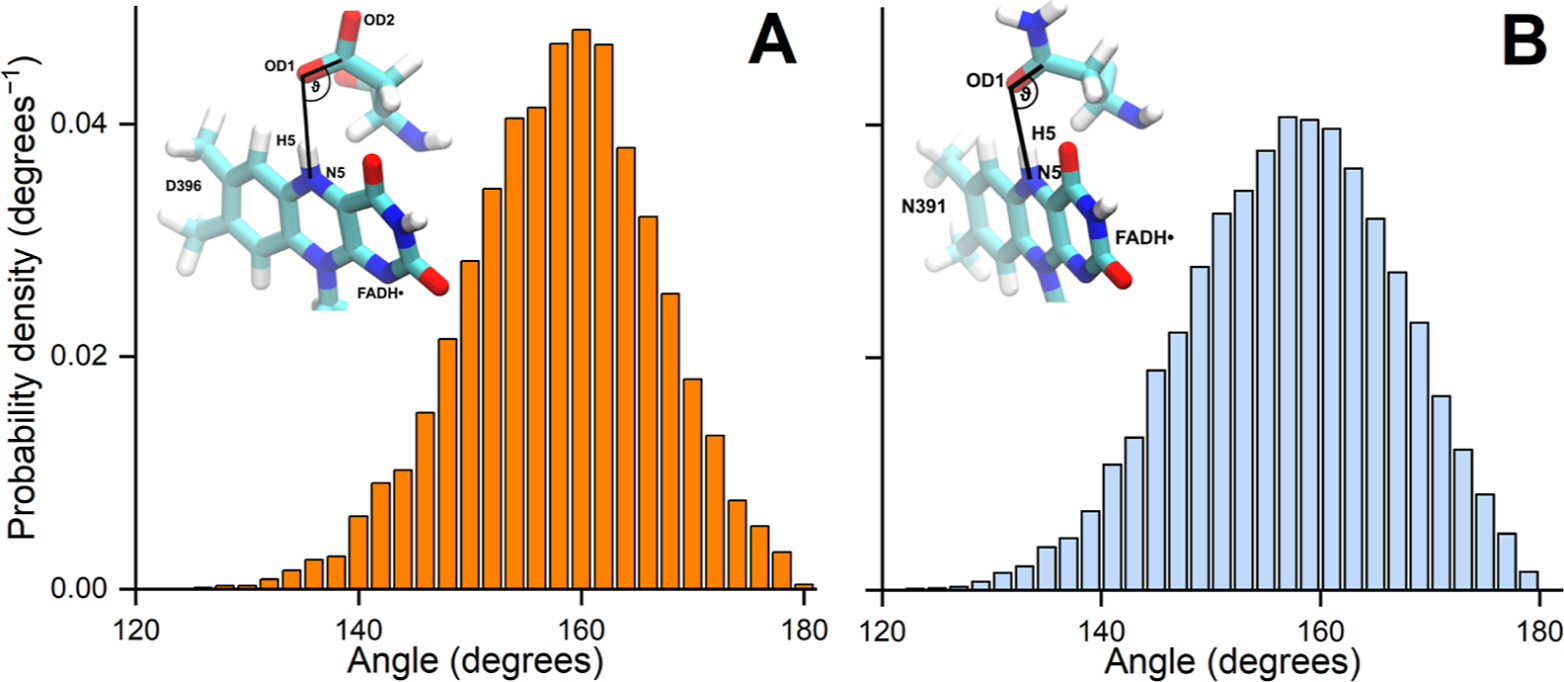
Normalized probability density distribution ρ(ϑ) of the sampled angles between the flavin part of the FADH^•^ cofactor (N5–H5 bond) and the OD1/OD2-C bond of the D396 residue in the AtCry1 protein (A) and the flavin part of the FADH^•^ cofactor (N5–H bond) and the OD1-C bond of the N391 residue in the ClCry4 protein (B).

To check whether an H-bond is indeed present, the angles between the flavin part of the FADH^•^ cofactor (N5 atom and bounded hydrogen atom) and the corresponding residue (D396 in AtCry1 and N391 in ClCry4) in both proteins have to be analyzed (see Figure 5). Normalized probability distributions for the computed hydrogen bonding angles in the AtCry1 protein (Figure 5A) prove the presence of an H-bond between the FADH^•^ cofactor and the D396 residue; the H-bond angle in this case is distributed at around 160°. In the ClCry4 protein, the probability distribution of the H-bond angle is similar and also peaks at around 160°. The angle involved in the H-bond formation is defined between the electronegative atom covalently bonded to the H-atom and another nearby electronegative atom. In the studied ClCry4 protein, this involves the N5 and H5 atoms from the FADH^•^ cofactor, along with the OD1 atom from the N391 residue (Figure 5A). Similarly, in the AtCry1 protein, the N5 and H5 atoms from the FADH^•^ cofactor form the angle with the OD1/OD2 atoms from the D396 residue (Figure 5B); here the closest oxygen atom to the H5 atom of the FADH^•^ cofactor is always considered. The influence of the H-bond and the molecular environment on the electronic structure of FADH^•^ is shown in the Supporting Information, by performing Mulliken charge analysis in the case of the two studied proteins, the difference of the partial charges of the nitrogen atoms of the flavin moiety is shown in Table S1, of the carbon atoms in Table S2 and of the oxygen atoms in Table S3. All tables show the standard deviation calculated when estimating the average of the partial atomic charges over the studied snapshots. The results indicate that the partial charges of the flavin moiety in ClCry4 are more influenced by the molecular environment than those in the case of the flavin moiety in the AtCry1 protein. This can be seen from the standard deviations of the average computed charges of especially the carbon atoms (see Table S2) where ClCry4 have consistently larger deviations from the average, compared to the analogous standard deviations computed for the atoms of the flavin moiety in the AtCry1 protein.

**Figure 6:**
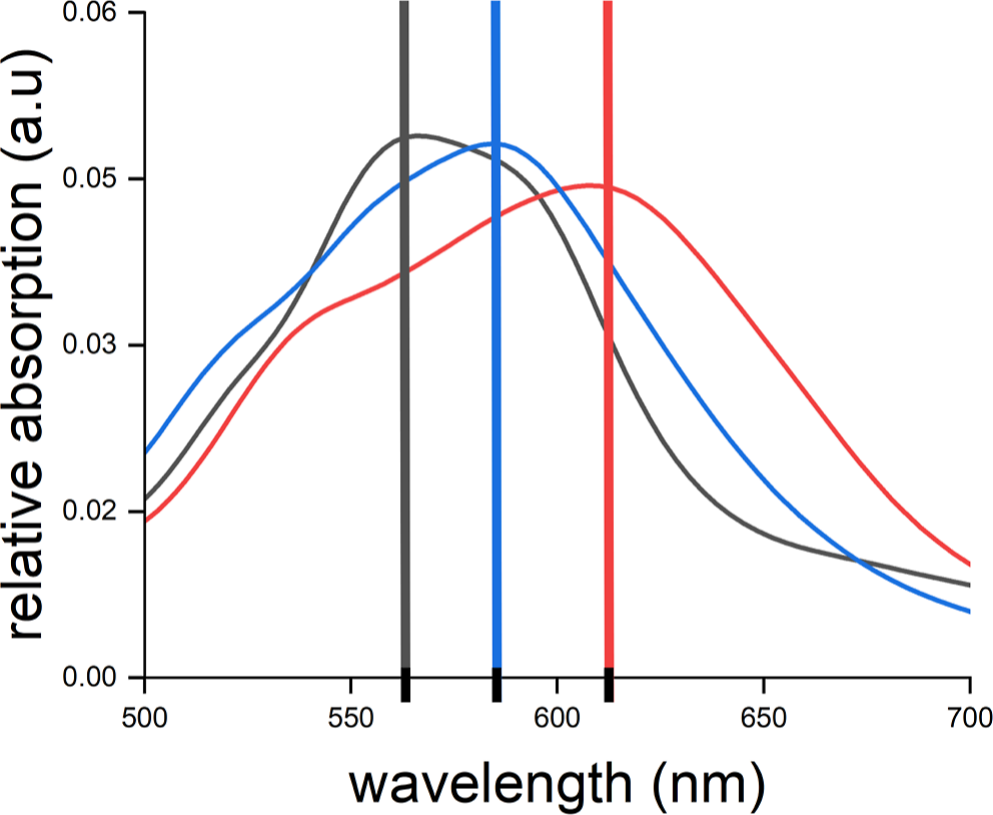
Averaged absorption spectra from the photoabsorption spectra computed for the FADH^•^ cofactor inside the ClCry4 protein: wild type (blue), excluded N391 residue (red), and N391D mutation (black). The peaks of the individual absorption spectra are highlighted here to illustrate the shift of the spectra in relation to each other. The spectra were recorded for a wavelength between 500 and 700 nm. All spectra from the individual snapshots are shown in Figure S1 in the Supporting Information.

The influence of the N391 residue on the absorption spectra of the ClCry4 protein was studied in further detail. For this purpose, the N391 residue in the ClCry4 protein was excluded in the spectra calculation to deduce whether the H-bond between the N391 residue and the FADH^•^ cofactor is responsible for the observed red shift. Additionally, the absorption spectra of the mutated ClCry4 protein were simulated again, where the N391 residue was replaced through an aspartic acid to see whether the red shift would still be present. The averaged absorption spectra resulting from all three calculations (wild type, excluded N391 residue, and N391D mutation) are shown in Figure 6, where the averaging was performed over a number of snapshots taken from the corresponding MD simulations (50 snapshots for the wild-type simulation and 15 snapshots for the N391D mutation). The absorption spectra at ∼600 nm for the three variants of ClCry4 reveal that the position of the peak varies depending on whether an H-bond is present or not. In the absence of an H-bond between the FADH^•^ cofactor and the N391 residue, the peak is red-shifted by ∼20 nm. Comparing the wild type and the N391D mutant shows that the peak is blue-shifted by ∼30 nm.

## Conclusions

The main goal of this study was to understand the red shift occurring in the experimentally obtained absorption spectrum of the FADH^•^ cofactor inside the ClCry4 protein.^22^ For this purpose, absorption spectra of the FADH^•^ cofactor were calculated by using the PE method.

MD simulations were carried out on the crystal structure of the ClCry4 protein with the FADH^•^ cofactor bound inside the protein. The equilibration simulation of the MD simulations revealed a notable displacement of the phosphate binding loop of the ClCry4 protein, consistent with the previous studies,^23^ while no notable changes were seen in the production simulation of the ClCry4 protein.

From the production simulation, 50 snapshots for the absorption spectra calculation of the FADH^•^ cofactor inside the ClCry4 protein were extracted. The absorption spectra obtained computationally were compared with the experimental absorption spectrum from the earlier studies,^22^ and an overall good agreement was demonstrated.

The absorption spectra of the ClCry4 protein were also compared to the calculated and experimental absorption spectra of the AtCry1 protein. A spectral red shift of the ClCry4 spectrum relative to the one recorded for the AtCry1 protein was found. The environment of the FADH^•^ cofactor was shown to be responsible for this difference in the absorption spectra. The N391 residue in the ClCry4 protein and the D396 residue in the AtCry1 protein were investigated in more detail since both residues in ClCry4 and AtCry1, respectively, might have an H-bond to the FADH^•^ cofactor. The spectral red shift could be attributed to a softening increase of the H-bond between the FADH^•^ cofactor and the N391 residue compared to the AtCry1 case, but more importantly, from the fact that the charges of heavy atoms of the flavin moiety in the ClCry4 protein vary more over different snapshots than in the AtCry1 protein. While the charge analysis partially answers the question of H-bond influence on the chromophore absorption properties in cryptochromes, it at the same time calls for a more in-depth analysis of the delicate polarization effects.

To pinpoint the influence of the H-bonding between the N391 residue and the FADH^•^ cofactor in ClCry4, the interaction with the N391 residue was removed in one simulation, and in another simulation, the N391 residue was replaced by an aspartic acid (D), since the FADH^•^ cofactor in the AtCry1 protein forms an H-bond with the D396 residue. The averaged absorption spectra were calculated for both simulations and compared with the averaged absorption spectrum for the wild-type ClCry4. The changes to the N391 residue red-shifted the absorption spectrum by 10–30 nm, which is in good agreement with a previous experimental study where a red shift in the range of ∼40 nm was observed.^22^

## Data Availability

The protein structure (PDB-ID: 6PU0—10.2210/pdb6PU0/pdb) modeled in this work was obtained from the RCSB protein databank.^22^ Simulations were performed using NAMD molecular dynamics software (http://www.ks.uiuc.edu/Research/namd/).^40,41^ Parameters for the CHARMM36 forcefield with CMAP corrections are available online (http://mackerell.umaryland.edu/charmm_ff.shtml).^32^ The VMD software (www.ks.uiuc.edu/Research/vmd/) was used to visualize molecular configurations and simulation trajectories and to create Figures 1, 4, and 5.^48^ To calculate the photoabsorption spectra, the DALTON program was utilized (https://daltonprogram.org/).^46^ To calculate the multipoles and polarizabilities, the LoProp approach was used (https://github.com/vahtras/loprop).^45^

## Supporting Information Available

Absorption spectra from the individual calculations (PDF)
