# Supplementary material for: Understanding the Red Shift in the Absorption Spectrum of the FAD Cofactor in ClCry4 Protein
Source: J Phys Chem B. 2024 May 28;128(22):5320–6. doi: 10.1021/acs.jpcb.4c00710 (PMC11163422; doi:10.1021/acs.jpcb.4c00710)
Supplement: Supplementary file 1 — jp4c00710_si_001.pdf [file jp4c00710_si_001.pdf]

# Understanding the redshift in the absorption spectrum of the FAD cofactor in ClCry4 protein (Supporting Information)

Katarina Kretschmer,[†] Anders Frederiksen,[†] Peter Reinholdt,[‡] Jacob Kongsted,[‡] and Ilia A. Solov'yov[*,†,¶,§]

†*Institute of Physics, Carl von Ossietzky Universität Oldenburg*
*Carl-von-Ossietzky Str. 9-11, 26129 Oldenburg, Germany*

‡*Department of Physics, Chemistry and Pharmacy, University of Southern Denmark*
*DK 5230-Odense, Denmark*

¶*Research Centre for Neurosensory Science, Carl von Ossietzky Universität Oldenburg*
*Carl-von-Ossietzky Str. 9-11, 26129 Oldenburg, Germany*

§*Center for Nanoscale Dynamics (CENAD), Carl von Ossietzky Universität Oldenburg*
*Ammerländer Heerstr. 114-118, 26129 Oldenburg, Germany*

E-mail: ilia.solovyov@uni-oldenburg.de

## Absorption spectra at the energy level

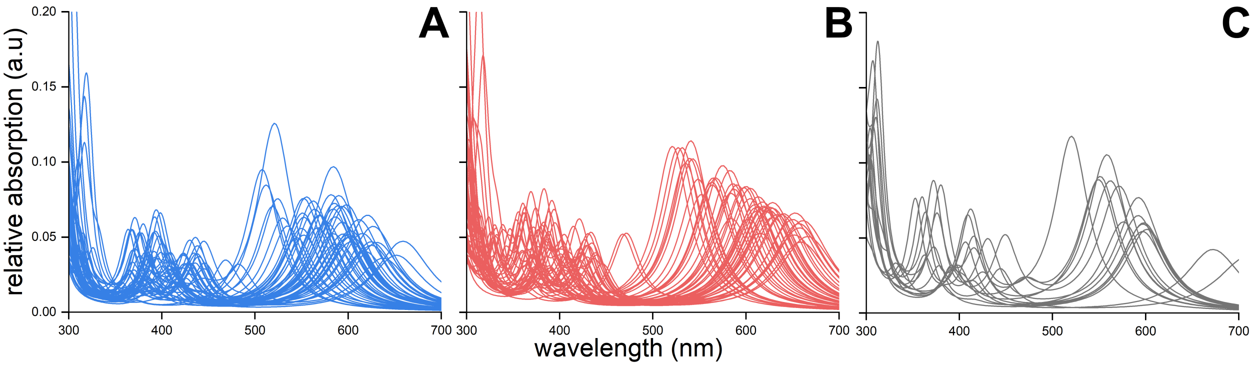


Figure S1: The averaged absorption spectra and their corresponding energies are shown for the FADH$^{\bullet}$ cofactor bound within the ClCry4 (red) and the AtCry1 protein (black). For both proteins, the average was calculated from all individual absorption spectra obtained from the snapshots of the system (Fig. 3). The vertical lines highlight an observed redshift in the spectral line of the FADH$^{\bullet}$ cofactor in the ClCry4 protein compared to the AtCry1 protein.

Figure S1 illustrates the computed photoabsorption spectrum of the FADH$^{\bullet}$ cofactor within the ClCry4 protein (red) plotted as a function of the excitation energies. The result is compared with the previously computed, averaged absorption spectrum at the respective energies of the FADH$^{\bullet}$ cofactor within the AtCry1 protein (black) reused from an earlier study.[1] Comparing the first peak of the averaged absorption spectra of the FADH$^{\bullet}$ cofactor within the ClCry4 protein with that within the AtCry1 protein suggests a redshift of $\sim 0.4$ eV. The first peak of the absorption spectrum for the ClCry4 protein (red vertical line) is found around 2.1 eV (equivalent to ∼590 nm), while for the AtCry1 (black vertical line), the first peak is seen at 2.5 eV (∼496 nm).

## Absorption spectra from the individual calculations

In Fig. S2 the obtained absorption spectra from all three MD simulations are shown. These absorption spectra from the respective calculations were used to determine the averaged absorption spectra shown in Fig. 6.

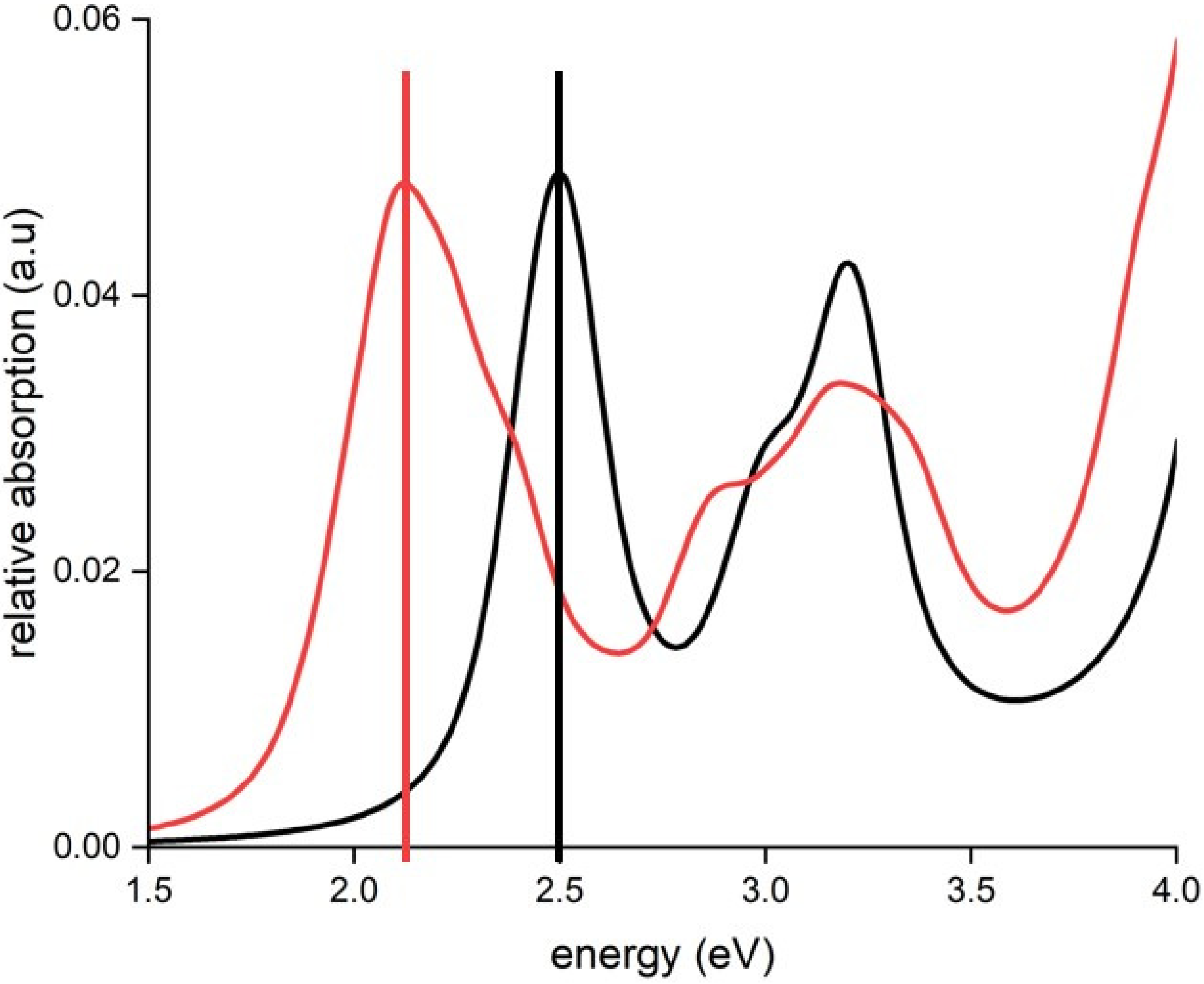


Figure S2: Absorption spectra of the $FADH^{\bullet}$ cofactor inside the ClCry4 protein from all individual calculations (wildtype (**A**), excluded interaction with the N391 residue (**B**), N391D mutation (**C**)). The absorption spectra are calculated from the MD snapshots taken from the three simulations.

# References

(1) Frederiksen, A.; Gerhards, L.; Reinholdt, P.; Kongsted, J.; Solov'yov, I. A. On the importance of polarizable embedding for photo-absorption spectrum calculations of Arabidopsis thaliana cryptochrome 1. **submitted**,

**Table S1:** Averaged charges of the nitrogen atoms from the flavin moitety of the FADH$^\bullet$ cofactor in AtCry1 and ClCry4. The charges correspond to the Mulliken charges and were averaged over the 51 snapshots used in the calculations. $\sigma$ denotes the corresponding standard deviation.

| | AtCry1 | | ClCry4 | |
|---|---|---|---|---|
| | Charge | $\sigma$ | Charge | $\sigma$ |
| N2 | -0.869 | ±0.036 | -0.856 | ±0.061 |
| N5 | -0.708 | ±0.030 | -0.653 | ±0.036 |
| N10 | -0.643 | ±0.039 | -0.590 | ±0.040 |
| N19 | 0.144 | ±0.048 | 0.114 | ±0.055 |

**Table S2:** Averaged charges of the carbon atoms of the flavin moitety from the FADH$^\bullet$ cofactor in AtCry1 and ClCry4. The charges correspond to the Mulliken charges and were averaged over the 51 snapshots used in the calculations. $\sigma$ denotes the corresponding standard deviation.

| | AtCry1 | | ClCry4 | |
|---|---|---|---|---|
| | Charge | $\sigma$ | Charge | $\sigma$ |
| C1 | -0.299 | ±0.135 | -0.342 | ±0.083 |
| C3 | 0.529 | ±0.064 | 0.527 | ±0.114 |
| C6 | 0.768 | ±0.010 | 0.722 | ±0.141 |
| C7 | 0.057 | ±0.102 | -0.090 | ±0.154 |
| C9 | 0.187 | ±0.124 | 0.426 | ±0.257 |
| C11 | -0.475 | ±0.163 | -0.697 | ±0.259 |
| C12 | 0.248 | ±0.275 | 0.334 | ±0.340 |
| C13 | -0.768 | ±0.083 | -0.885 | ±0.178 |
| C14 | 0.265 | ±0.265 | 0.078 | ±0.437 |
| C15 | -0.854 | ±0.073 | -0.999 | ±0.168 |
| C16 | -0.409 | ±0.212 | -0.189 | ±0.302 |
| C17 | 0.355 | ±0.232 | 0.503 | ±0.377 |
| C18 | 0.342 | ±0.090 | 0.473 | ±0.154 |

**Table S3:** Averaged charges of the oxygen atoms from the flavin moitety of the FADH$^\bullet$ cofactor in AtCry1 and ClCry4. The charges correspond to the Mulliken charges and were averaged over the 51 snapshots used in the calculations. $\sigma$ denotes the corresponding standard deviation.

| | AtCry1 | | ClCry4 | |
|---|---|---|---|---|
| | Charge | $\sigma$ | Charge | $\sigma$ |
| O4 | -0.636 | 0.039 | -0.638 | 0.049 |
| O8 | -0.635 | 0.055 | -0.597 | 0.040 |
